# Optimization of Hydrogen Peroxide Detection for a Methyl Mercaptan Biosensor

**DOI:** 10.3390/s130405028

**Published:** 2013-04-15

**Authors:** Zhan-Hong Li, Houssemeddine Guedri, Bruno Viguier, Shi-Gang Sun, Jean-Louis Marty

**Affiliations:** 1 State Key Laboratory of Physical Chemistry of Solid Surfaces, and Department of Chemistry, College of Chemistry and Chemical Engineering, Xiamen University, Xiamen 361005, China; E-Mails: lzh1508@gmail.com (Z.-H.L.); sgsun@xmu.edu.cn (S.-G.S.); 2 Laboratoire IMAGES EA 4218, Groupe Biocapteurs, Université de Perpignan Via Domitia (UPVD), Bâtiment S, 52 av Paul Alduy 66860 Perpignan Cedex, France; E-Mails: houssemeddine.guedri@live.fr (H.G.); viguierbr@gmail.com (B.V.)

**Keywords:** methyl mercaptan, hydrogen peroxide, amperometric sensor, screen printed electrode

## Abstract

Several kinds of modified carbon screen printed electrodes (CSPEs) for amperometric detection of hydrogen peroxide (H_2_O_2_) are presented in order to propose a methyl mercaptan (MM) biosensor. Unmodified, carbon nanotubes (CNTs), cobalt phthalocyanine (CoPC), Prussian blue (PB), and Os-wired HRP modified CSPE sensors were fabricated and tested to detect H_2_O_2_, applying a potential of +0.6 V, +0.6 V, +0.4 V, −0.2 V and −0.1 V (*versus* Ag/AgCl), respectively. The limits of detection of these electrodes for H_2_O_2_ were 3.1 μM, 1.3 μM, 71 nM, 1.3 μM, 13.7 nM, respectively. The results demonstrated that the Os-wired HRP modified CSPEs gives the lowest limit of detection (LOD) for H_2_O_2_ at a working potential as low as −0.1 V. Os-wired HRP is the optimum choice for establishment of a MM biosensor and gives a detection limit of 0.5 μM.

## 1. Introduction

Methyl mercaptan (MM) is one of the volatile sulfur compounds (VSCs), which are known to be involved in halitosis (bad breath) [1,2] and periodontal diseases [2], and the predominant causative factor of noticeable oral malodor [3]. MM is also present in several other cases such as the bottle storage of wines [4]; wood-pulp mills, sewage treatment plants and factories producing jet fuel, pesticides and plastics [5]; and even in the atmosphere and on the ocean surface [6]. Consequently, MM detection is important in the dental, medical, food, environment and atmosphere fields.

A low-cost, sensitive and specific sensor for detecting MM could be an interesting alternative to conventional MM monitoring methods such as the use of a halimeter, an expensive device, in the dental field [7,8]. Biosensors to monitor MM have been described by Mitsubayashi *et al*. [9–12]. In their work, monoamine oxidase A (MAO-A) or flavin-containing monooxygenase (FMO) was used to catalytically oxidize MM, and the oxygen consumption induced by this reaction was monitored. Coupled with this system, a substrate regeneration cycle with ascorbic acid was carried out. However, a sensor for the detection of O_2_ depletion, which has the initially high current background of the oxygen electrode [13], is less sensitive than one for H_2_O_2_ measurement. To solve this problem and seek sensitive detection methods, our objective was to develop a MM biosensor coupled with sensitive hydrogen peroxide detection. Alcohol oxidase (AOX) is known to catalytically oxidize MM with production of formaldehyde, sulfide and H_2_O_2_ [14] according to the reaction:

$${\text{H}}_{3}\text{C}\;‐\;\text{SH}+{\text{O}}_{2}+{\text{H}}_{2}\text{O}\overset{\left[\text{AOX}\right]}{\to }{\text{H}}_{2}\text{C}\;=\;\text{O}+{\text{H}}_{2}\text{S}+{\text{H}}_{2}{\text{O}}_{2}$$

Hydrogen peroxide generated during enzyme-catalyzed reactions can be electrochemically detected on modified/unmodified carbon matrixes [15–27]. In this work, unmodified carbon nanotube (CNT), cobalt phthalocyanine (CoPC), Prussian blue (PB), and Os-wired HRP modified screen printed electrode (CSPE) sensors were fabricated and tested to detect H_2_O_2_. Our aim was to seek the most sensitive and optimal detection method of H_2_O_2_ for a MM amperometric biosensor.

## 2. Experimental

### 2.1. Reagents

Hydrogen peroxide (H_2_O_2_, 30%, w/w), disodium hydrogen phosphate (Na_2_HPO_4_), potassium chloride (KCl), sodium chloride (NaCl), acetic acid (CH_3_COOH), sodium acetate trihydrate (CH_3_COONa·3H_2_O), potassium hexacyanoferrate (III) (K_3_Fe(CN)_6_), *o*-phenylenediamine (99.5%), bovine serum albumin (BSA, ≥96%) were obtained from Sigma-Aldrich (Lyon, France). The concentration of diluted H_2_O_2_ solutions was determined by the classic potassium permanganate titration method. Sulfuric acid (95%) (H_2_SO_4_) and NaH_2_PO_4_·2H_2_O were purchased from Prolabo (Briare, France). Hydrochloric acid (HCl) (37%) was obtained from Carlo Erba Reagenti (Milan, Italy). Peroxidase redox polymer (Os-wired HRP) was purchased from Bioanalytical Systems, Inc. (Gloucestershire, UK). Carbon pastes used for screen printed electrodes (Electrodag PE-410, 423SS and 6037SS) were obtained from Acheson (Plymouth, UK). A glycerolphtalic paint (Astral, France) was used as insulating layer. Transparent PVC sheets (200 mm × 100 mm × 0.5 mm) (SKK, Denzlingen, Germany) were used as screen-printing substrates. All chemicals were used without any further purification. All solutions were prepared using Milli-Q water.

### 2.2. Instrument

**Figure 1.**
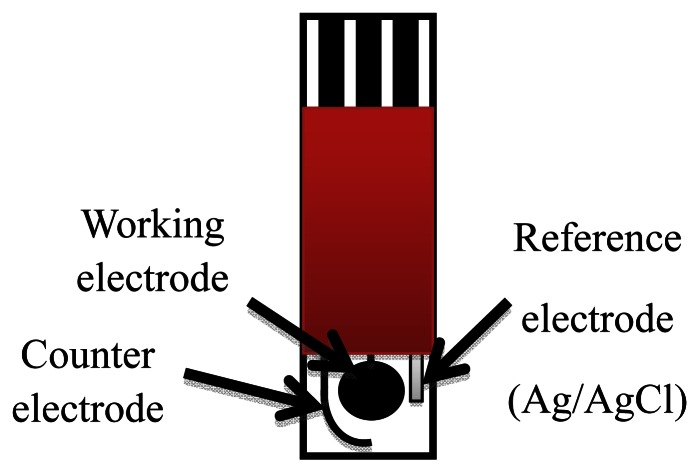
Screen printed electrode.

CSPEs were produced in the laboratory using a semi-automatic DEK 248 screen-printing system (DEK, Weymounth, UK). The working electrode was a 4 mm diameter disk, the auxiliary electrode was a 16 mm × 1.5 mm curved wire and the Ag/AgCl reference electrode was a 5 mm × 1.5 mm straight wire (Figure 1).

Cyclic voltammetry (CV) measurements, PB electrodeposition, PPD electropolymerisation and amperometric measurements were carried out on an AUTOLAB PGSTAT100 (Metrohm, Switzerland), using GPES v4.7 (Metrohm) as informatic interface. All potential values are reported *versus* Ag/AgCl. Amperometric measurements were performed in a 10 mL glass bath cell with magnetic stirring at room temperature.

### 2.3. Carbon Screen Printed Electrode Modifications

#### 2.3.1. Preparation of CNT Modified CSPEs

CNT modified CSPEs were prepared as described in the work of Silveira *et al*. [28]. Briefly, 10 μL of 0.3 mg/mL SWCNT water dispersion were successively deposited on the CSPEs working electrode, drying each layer one by one under vacuum. The electrodes were then washed with water.

#### 2.3.2. Preparation of CoPC Modified CSPEs

Cobalt-phtalocyanine-modified paste was purchased from Gwent Electronic Materials, Ltd. (Gwent, UK) and modifications were performed on working electrode by the DEK screen-printing system.

#### 2.3.3. Preparation of PB/PPD Modified CSPEs

The PB film was first deposited by covering the CSPEs with a solution containing 2.5 mM FeCl_3_, 2.5 mM K_3_Fe(CN)_6_, 0.1 M KCl and 0.1 M HCl and applying a potential +0.4 V *versus* Ag/AgCl for 40 s. Then the PB film was activated by covering the electrode by a solution containing 0.1 M KCl and 0.1 M HCl, electrochemically cycling for 20 cycles between −0.05 V and 0.35 V *versus* Ag/AgCl at a scan rate of 50 mV·s^−1^. After washing with distilled water, it was dried for 1 h at 100 °C in oven. To improve the stability and selectivity properties of the PB electrodes, the electropolymerisation of a poly- (*o*-phenylenediamine) (PPD) coat was formed. The PPD layer was deposited by electrochemically cycling the PB modified electrode with potential between −0.5 V and 0.7 V *versus* Ag/AgCl at a scan rate of 50 mV·s^−1^ in deaerated 0.1 M, pH 5.0 acetate buffer solution containing 0.5 mM *o*-phenylenediamine under a stream of nitrogen [29].

#### 2.3.4. Preparation of Os Wired HRP (Os-HRP) Modified CSPEs

For the Os-HRP modified CSPEs, 10 μL 0.1 M phosphate buffer solution, pH 7.5, containing 10% (v/v) Os-HRP was deposited on the surface of CSPEs. It was allowed to dry at room temperature for 2 h. It was thoroughly washed with buffer before use.

## 3. Results and Discussion

### 3.1. H_2_O_2_ Detection with Unmodified CSPEs

**Figure 2.**
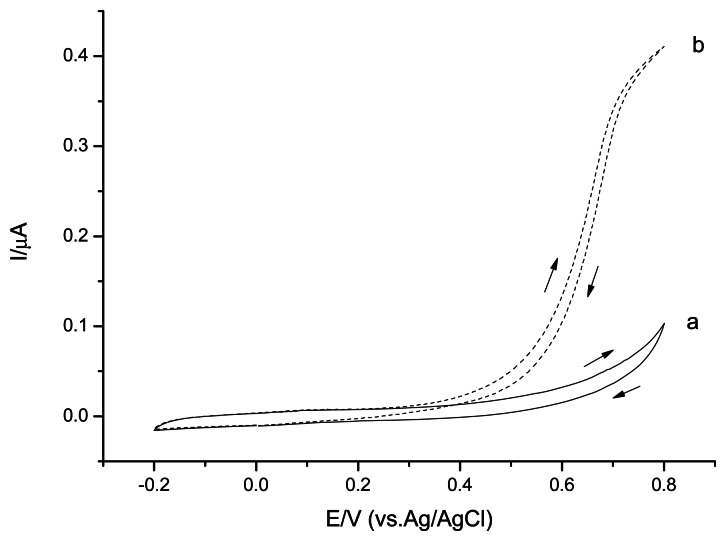
Cyclic voltammograms of CSPEs in 0.1 M phosphate buffer solution, pH 7.5 (a); in presence of 1 mM H_2_O_2_ (b). Scan rate 20 mV·s^−1^.

The cyclic voltammetry studies were performed in 0.1 M phosphate buffer solution, pH 7.5 to investigate the CSPEs' electrochemical behavior (Figure 2). CSPEs showed no obvious peak in the absence of H_2_O_2_ in the potential range from −0.2 V∼0.8 V *versus* Ag/AgCl. In the presence of H_2_O_2_, CSPEs started to perform current response at potential around +0.3 V *versus* Ag/AgCl, indicating the onset potential of the H_2_O_2_ electrooxidation.

**Figure 3.**
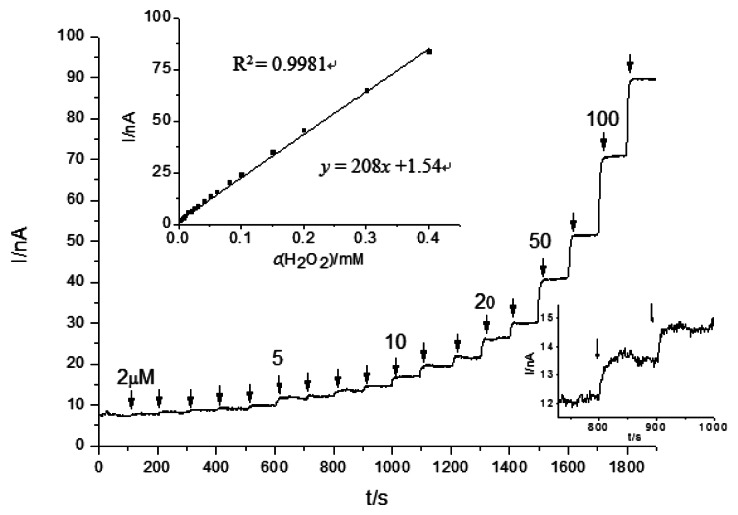
Amperometric performance of CSPEs for detection of H_2_O_2_ at +0.6 V in a stirred 0.1 M phosphate buffer solution, pH 7.5. The upper left inset shows the calibration curve of CSPEs for H_2_O_2_ concentration; the lower right inset zooms in the part of the amperometric response of 5 μM H_2_O_2_ injected.

For the investigation of H_2_O_2_ limit detection, chronoamperometry experiments were carried out with several concentrations of H_2_O_2_ injected into the stirred bath cell (Figure 3). For unmodified CSPEs, the H_2_O_2_ detection limit was 3.1 μM (S/N = 3) applying a +0.6 V potential *versus* Ag/AgCl, and the current response slope of the calibration curve was 0.208 μA/mM. To investigate the reproducibility, three parallel measurements with 0.1 mM H_2_O_2_ revealed a relative standard deviation (RSD) of 12.1%. The high RSD observed of unmodified CPSEs is likely related to the marked differences in the real active electrode area, which is difficult to handle and adjust.

### 3.2. H_2_O_2_ Detection with CNT/CSPEs

**Figure 4.**
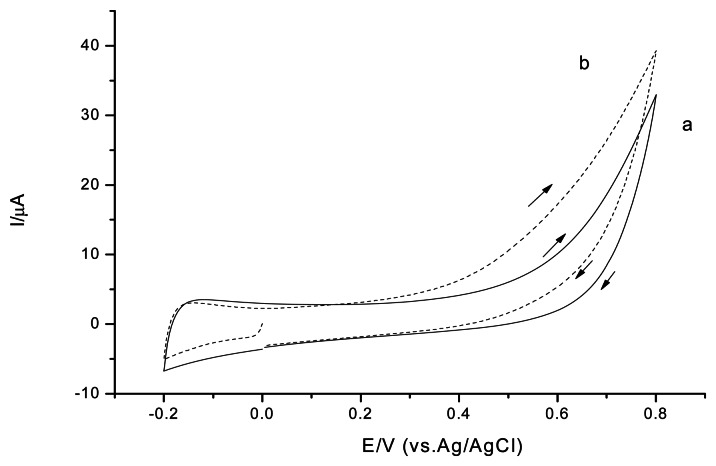
Cyclic voltammograms of CNT/CSPEs in 0.1 M phosphate buffer solution, pH 7.5 (a); in presence of 1 mM H_2_O_2_ (b). Scan rate 20 mV·s^−1^.

Both CSPEs (Figure 2) and CNT/CSPEs (Figure 4) showed a H_2_O_2_ oxidation peak in the cyclic voltammetry experiments for the studied potential range. The onset potential of the H_2_O_2_ electrooxidation for CNT/CSPEs was around +0.2 V *versus* Ag/AgCl, the detection limit was 1.3 μM (S/N = 3) applying a positive potential of +0.6 V *versus* Ag/AgCl, and the current response slope of CNT/CSPEs for H_2_O_2_ was 32.1 μA/mM. To investigate the reproducibility, three parallel measurements with 0.1 mM H_2_O_2_ revealed a RSD of 19.7%.

Both unmodified CSPEs and CNT are intrinsically carbon. Compared to unmodified CSPEs, detections of H_2_O_2_ for CNT/CSPEs need lower oxidation potential (CSPEs, +0.3 V; CNT/CSPEs, +0.2 V), and have lower detection limit (CSPEs, 3.1 μM; CNT/CSPEs, 1.3 μM) with higher current response (CSPEs, 0.208 μA/mM; CNT/CSPEs, 32.1 μA/mM). The increased current response may arise from the large electric active area and a thin, porous diffusion layer [30]; the reasons of lower onset oxidation potential and lower detection limit are still controversial [31], because CNT may contain metal impurities derived from the catalysts used for their growth [32,33]. In a sense, CNT/CSPEs could be more favorable than unmodified CSPEs for H_2_O_2_ detection.

### 3.3. H_2_O_2_ Detection with CoPC/CSPEs

**Figure 5.**
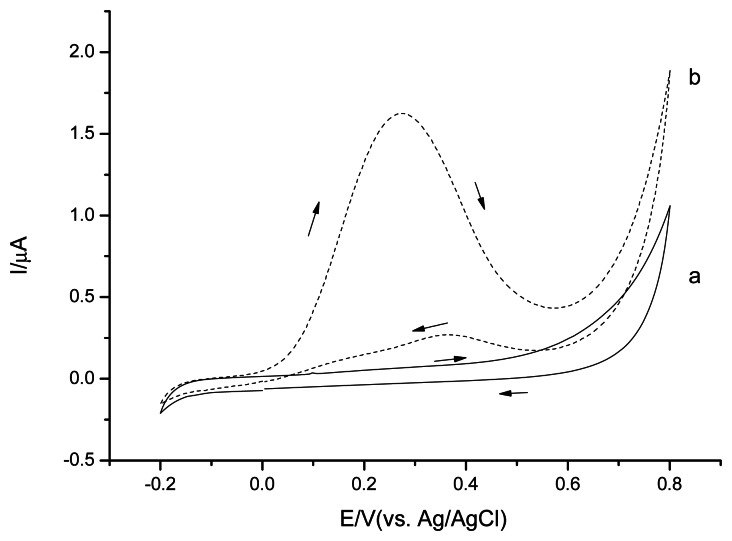
Cyclic voltammograms of CoPC/CSPEs in 0.1 M phosphate buffer solution, pH 7.5 (a); in presence of 0.1 mM H_2_O_2_ (b). Scan rate 20 mV·s^−1^.

To investigate the electrochemical behavior of CoPC/CSPEs in phosphate buffer solution, cyclic voltammetry experiments were carried out in the potential range of −0.2 V∼0.8 V *versus* Ag/AgCl (Figure 5). The presence of a well-defined oxidation current peak at around +0.3 V *versus* Ag/AgCl is consistent with the following reaction [21]:

$$\begin{array}{c} 2{\text{Co}}^{2+}+{\text{H}}_{2}{\text{O}}_{2}\to 2{\text{CO}}^{+}+{\text{O}}_{2}+2{\text{H}}^{+}\\ 2{\text{Co}}^{+}\to 2{\text{Co}}^{2+}+2{\text{e}}^{-} \end{array}$$

The reaction can be described by a chemical-electrochemical (CE) mechanism [21]: H_2_O_2_ chemically reduces Co^2+^ to Co^+^ and its subsequent electrochemical re-oxidation is observed as an oxidation peak. Consequently, this peak is used for the quantification of H_2_O_2_. For CoPC/CSPEs, the H_2_O_2_ detection limit was calculated as 71 nM (S/N = 3) applying a positive potential of +0.4 V *versus* Ag/AgCl in chronoamperometry experiments, and the slope of the calibration curve was 3.7 μA/mM by. Three parallel measurements with 10 μM H_2_O_2_ reveal a RSD of 2.4%, indicating a good reproducibility for this sensor.

### 3.4. H_2_O_2_ Detection with PPD/PB/CSPEs

PB is known to have a high solubility in neutral and basic solutions [34,35], consequently the PB-modified electrodes may have stability problems under our conditions. To improve the stability and selectivity of the PB electrodes a PPD coat is formed. In order to assess the effect of the PPD coat on the stability of the PB modified electrodes, cyclic voltammetry experiments were carried out in 0.1 M phosphate buffer pH 7.5 (not shown in this paper).

**Figure 6.**
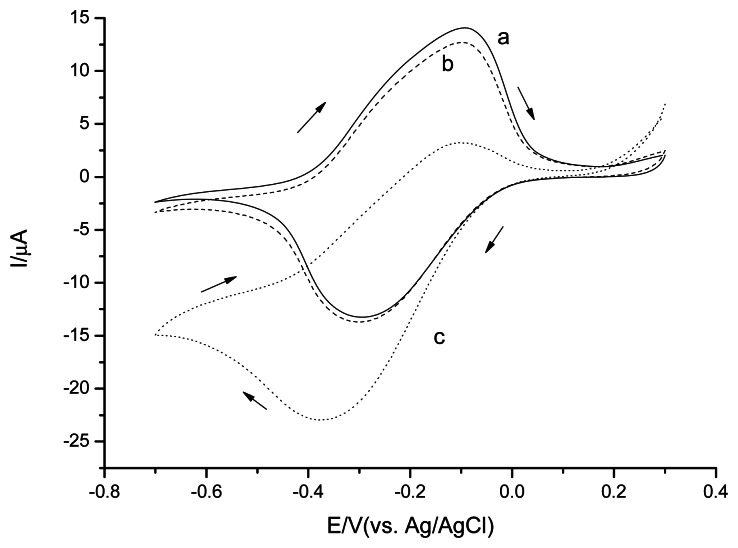
Cyclic voltammograms of PPD/PB/CSPEs in 0.1 M phosphate buffer solution, pH 7.5 (a); in presence of 0.1 mM H_2_O_2_ (b) and 1 mM H_2_O_2_ (c). Scan rate 20 mV·s^−1^.

Without PPD layer coating, PB dissolved in solution, resulting in a CV current decrease in the scanning process. After 20 scan cycles, the PB/CSPEs CV's performance were similar to the unmodified CSPEs (Figure 2). With PPD layer coating, the response of redox peaks of PB reduced slightly even after 50 scan cycles, indicating that the PPD layer stabilized the PB. To investigate the electrochemical performance of PPD/PB/CSPEs H_2_O_2_ was added to the solution (Figure 6).

In the phosphate solution, the redox peaks correspond to the reduction of Prussian blue and oxidation of Prussian white as [36]:

$$\begin{array}{c} {{\text{Fe}}_{4}}^{\left(\text{III}\right)}{\left[{\text{Fe}}^{\left(\text{II}\right)}{\left(\text{CN}\right)}_{6}\right]}_{3}+{\text{K}}^{+}+{\text{e}}^{-}↔{\text{K}}_{4}{{\text{Fe}}_{4}}^{\left(\text{II}\right)}{\left[{\text{Fe}}^{\left(\text{II}\right)}{\left(\text{CN}\right)}_{6}\right]}_{3}\\ \begin{array}{cc} \;\text{Prussian blue} & \;\text{Prussian white} \end{array} \end{array}$$

In the presence of H_2_O_2_, the electrocatalytical reductive reaction of PB towards to H_2_O_2_ can be described as:

$$\begin{array}{c} {\text{K}}_{4}{{\text{Fe}}_{4}}^{\left(\text{II}\right)}{\left[{\text{Fe}}^{\left(\text{II}\right)}{\left(\text{CN}\right)}_{6}\right]}_{3}+{\text{H}}_{2}{\text{O}}_{2}\to {{\text{Fe}}_{4}}^{\left(\text{III}\right)}{\left[\text{F}{\text{e}}^{\left(\text{II}\right)}{\left(\text{CN}\right)}_{6}\right]}_{3}+{\text{K}}^{+}+\text{O}{\text{H}}^{-}\\ \begin{array}{cc} \text{Prussian white} & \;\text{Prussian blue} \end{array} \end{array}$$

For PPD/PB/CSPEs, the H_2_O_2_ detection limit was 1.3 μM (S/N = 3) for a cathode potential of −0.2 V and the current response slope was −33 μA/mM. Three parallel measurements with 10 μM H_2_O_2_ revealed a RSD = 4.1%. The lower RSDs observed of CoPC/CSPEs and PPD/PB/CSPEs than for unmodified CPSEs and CNT/CSPEs are likely related to the better control of mediator deposition.

### 3.5. H_2_O_2_ Detection with Os-HRP/CSPEs

**Figure 7.**
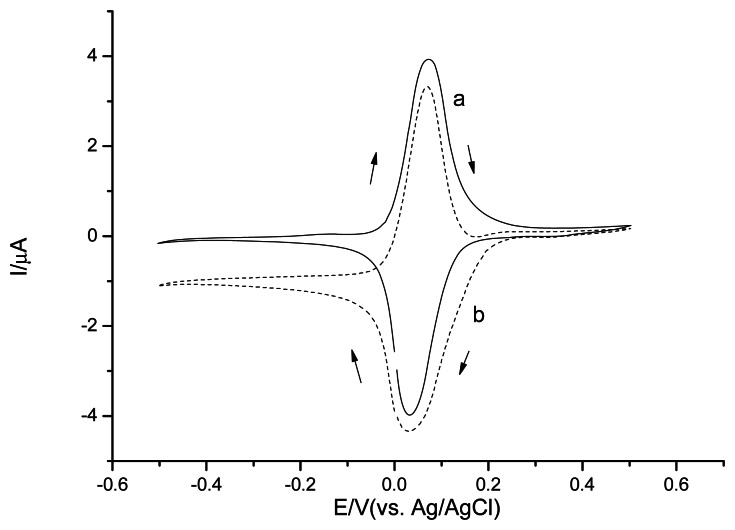
Cyclic voltammograms of Os-HRP/CSPEs in 0.1 M phosphate buffer solution, pH7.5 (a____); in presence of 0.1 mM H_2_O_2_ (b-----). Scan rate 20 mV·s^−1^.

**Scheme 1.**
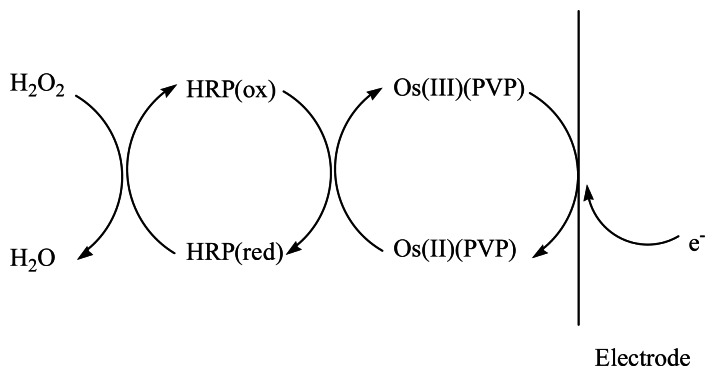
The electrocatalytical reductive reaction process of Os-HRP towards H_2_O_2_.

The Os-HRP/CSPEs show a couple of stable and well-defined redox peaks at around +30 mV and +70 mV at a scan rate of 20 mV·s^−1^ (Figure 7 curve a). In the presence of H_2_O_2_ (Figure 7 curve b), the electrocatalytical reductive reaction process of Os-HRP towards to H_2_O_2_ can be described by Scheme 1.

**Figure 8.**
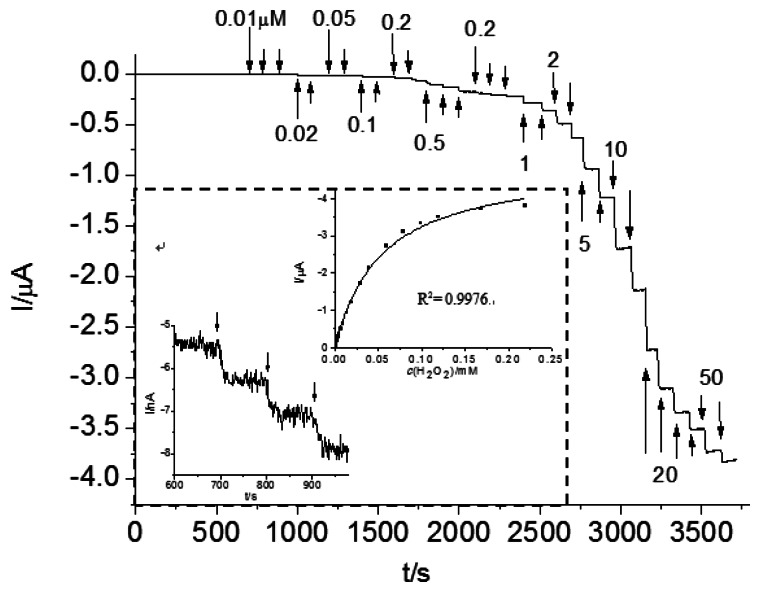
Amperometric performance of Os-HRP/CSPEs sensor in detection of H_2_O_2_ at −0.1 V in a stirred 0.1 M phosphate buffer solution, pH 7.5. The upper right inset shows the calibration curve of Os-HRP/CSPEs for H_2_O_2_ concentration; the lower left inset zooms in on the part of the amperometric response of 0.01 μM H_2_O_2_ injected.

For Os-HRP/CSPEs, the H_2_O_2_ detection limit was 13.7 nM (S/N = 3) at a cathode potential −0.1 V *versus* Ag/AgCl (Figure 8). According to the Michaelis-Menten equation, the calculated apparent K_M,p_, from the curve fitting is 53.5 μM. This low K_M,p_ value indicates Os-HRP's high affinity and high effective conversion for the H_2_O_2_ substrate and a favorable electron-transfer rate with the osmium mediator. A linear range was obtained until 25 μM. Three parallel measurements with 1 μM H_2_O_2_ revealed a RSD of 1.3%, which indicates a good reproducibility. This may be ascribed to the application of a low cathode potential to avoid the interferences with electro-active species. In addition, Os-HRP/CSPEs can be the most specific in detection of H_2_O_2_ because of the specificity of the reaction between HRP and H_2_O_2_.

The comparison of the amperometric analytical behavior to H_2_O_2_ of the five kinds of electrodes developed in this study is summarized in Table 1. It shows that the Os-HRP/CSPEs are the most sensitive electrodes with a low reduction potential applied for H_2_O_2_ detection. This is due to the specific, sensitive and rapid turnover of Os-HRP to H_2_O_2_.

Interference of electro-active species [25,37–39] is often encountered when using amperometric biosensors and applying a high potential in real samples. The decrease of the applied potential can be effective to avoid a lot of electrochemical interferences. With this consideration, PPD/PB/CSPEs and Os-HRP/CSPEs are used to combine with alcohol oxidase (AOX) in bovine serum albumin matrix to detect methyl mercaptan (MM) applying a low potential in the aqueous phase. The limit of detection of AOX/PPD/PB/CSPEs to MM is 10 μM; of AOX/Os-HRP/CSPEs to MM is 0.5 μM. For AOX/Os-HRP/CSPEs, the calibration curve of the response to MM is linear in the concentration range 0∼15 μM with a good correlation with the classical analytical method. We are working on the stability of the biosensor which is the crucial point to improve its accuracy and reliability. Consequently, Os-HRP/CSPEs are combined with alcohol oxidase an optimum method which gives the best sensitivity in methyl mercaptan detection.

## 4. Conclusions

Five kinds of modified carbon screen printed electrodes applied for H_2_O_2_ amperometric detection for MM biosensors were presented in this work. In comparison, and despite a worse reproducibility, CNT/CSPEs are a better choice than unmodified CPSEs in H_2_O_2_ detection resulting from their lowest detection limit, lowest onset oxidation potential and highest current response of CNT/CSPEs. However, the applied potential of +0.6 V *versus* Ag/AgCl is too positive to avoid the interference of electro-active species. CoPC/CSPEs and PPD/PB/CSPEs are also a good choice in H_2_O_2_ detection because of their low applied potential, low detection limit and good reproducibility. Os-HRP/CSPEs display the lowest detection limit and the best operational reproducibility towards H_2_O_2_. With the cathode potential applied and the use of HRP, Os-HRP/CSPEs can avoid the interference of electro-active species and be specific for H_2_O_2_ detection. The Os-wired HRP modified screen printed electrode is the optimum method we used to combine with alcohol oxidase in a methyl mercaptan biosensor, usable in both aqueous and gaseous phase detection.

## Figures and Tables

**Table 1.** Comparison of the amperometric analytical performances for H_2_O_2_ detection with the five kinds of electrodes prepared in the present work.

**Type of Electrodes**	**Potential Applied (V)**	**LOD (S/N = 3)**	**RSD (Tested Concentration of H_2_O_2_)**
CSPEs	+0.6	3.1 μM	12.1% (0.1 mM)
CNT/CSPEs	+0.6	1.3 μM	19.7% (0.1 mM)
CoPC/CSPEs	+0.4	71 nM	2.4% (10 μM)
PPD/PB/CSPEs	−0.2	1.3 μM	4.1% (10 μM)
Os-HRP/CSPEs	−0.1	13.7 nM	1.3% (1 μM)
